# 

**Published:** 1880-09-20

**Authors:** 


### TABLE OF CONTENTS.

ORIGINAL AND SELECTED ARTICLES.

Hydroleine—Hydrated Oil, by J. H. Egan, M.D., Tenn., 313. Gleanings from Private Practice, by C. C. Vanberbeck, M.D., PH. D., N. J., 315. Clinical Lecture, by H. A. Wilson, M.D., Philadelphia, 320. Production of Sex at Will, 323. How to Prevent Mammary Abscess, by S. S. Boyd, M.D., 326. Restoration after the Hand is completely Separated from the Arm, by L. .L Staton, M.D., N. C., 327. Domestic Remedy for Felons, by T. C. Brannon, M.D., Texas, 328. Contraction of Fingers, 329.

ABSTRACTS AND GLEANINGS.

Dr. Tanner's Fast, 330. The Effects of Conception on the Nervous System, 331. Leucocythæmia, 332. Consumption on the Salisbury Plan, 334. Analysis of a Secret Cancer Plaster, 335. The Use of Quinine in Connection with Nervous Sedatives, 336. Prostration and Nervousness after Coi[illegible]ion, 337. Sciatica of Rheumatic Origin; For Arresting Gonorrhea, 338. Milk and Lime Water; Strychnia Successfully Antidoted by Hydrate of Chloral, 359. Iodoform in Otorrhœa; A New Anthelmintic; Metaphosphoric Acid, 340. Nutritive Enemata; Benzoic Acid in Rheumatism; Iron on Digestion, 341. Distinguishing Central from Peripheral Paralysis of the Face; Saving Time; A Substitute for Quinine; Jaborandi in Mumps; Caution in regard to Chrysophanic Acid, 342.

SCIENTIFIC ITEMS.

Why we are Right-Handed; Treatment of Stammering, 343. Photography Under Water; Telephone; A Winking Portrait, 344.

PRACTICAL NOTES AND FORMULÆ.

Wine of Tar for Chronic Coughs; To Disguise Castor Oil; Saint Barthelemy's Fever Liniment; Benzoate of Soda, 345. Diphtheria; Hæmoptysis; Hoof Ointment, 346. Artificial Selter's Water; A Palatable Solution of Salicylic Acid; Arsenic in Neuralgia; Aristocratic Remedy for Itch, 347. Dyspepsia and Consumption; For the Removal of Tan and Freckles; Simple Elixir; For Barber's Itch; For Phthisis; Eucalyptol as an Antiseptic, 348.

EDITORIAL AND MISCELLANEOUS.

Editorial Notices, 349. Book Notices, 350. Special Notices, 322.

